# Pulsatile nanofluid flow with variable pressure gradient and heat transfer in wavy channel

**DOI:** 10.1038/s41598-024-59251-9

**Published:** 2024-04-23

**Authors:** A. S. Dawood, Faisal A. Kroush, Ramzy M. Abumandour, Islam M. Eldesoky

**Affiliations:** 1 Basic Engineering Sciences Department, Faculty of Engineering, Menofia University, Shebin El-Kom, 32513 Egypt; 2 Dean of Menofia Higher Institute of Engineering and Technology, El-Bagour, 32829 Egypt

**Keywords:** Pulsatile flow, Nanofluid, Magnetic field, Heat transfer, Perturbation method, Applied mathematics, Engineering

## Abstract

This research contributes to the comprehension of nanofluid behaviour through a wavy channel, emphasizing the significance of considering diverse influences in the modelling process. The study explores the collective influence of pressure gradient variation, magnetic field, porosity, channel waviness, nanoparticle concentration, and heat transfer on nano-blood flow in a two-dimensional wavy channel. In contrast to prior research assuming a constant pulsatile pressure gradient during channel waviness, this innovative study introduces a variable pressure gradient, significantly influencing several associated parameters. The mathematical model characterizing nano-blood flow in a horizontally wavy channel is solved using the perturbation technique. Analytical solutions for fundamental variables such as stream function, velocity, wall shear stress, pressure gradient, and temperature are visually depicted across different physical parameters values. The findings obtained for differing parameter values in the given problem demonstrate a significant influence of the amplitude ratio parameter of channel waviness, Hartmann number of the magnetic field, permeability parameter of the porous medium, volume fraction of nanoparticles, radiation parameter, Prandtl number, and the suction/injection parameter on the flow dynamics. The simulations provide valuable insights into the decrease in velocity with increasing magnetic field and its increase with higher permeability. Additionally, the temperature is observed to escalate with a rising nanoparticle volume fraction and radiation parameter, while it declines with increasing Prandtl number.

## Introduction

Understanding fluid dynamics in the context of blood flow through a wavy channel is highly significant in cardiovascular disease research. One critical application is biomedical engineering, particularly in understanding blood flow behaviour in microvascular systems. By analysing the flow of nano-blood with considerations for a magnetic field and heat transfer, this model could offer insights into blood flow dynamics in vessels subjected to magnetic fields, such as those used in targeted drug delivery systems or magnetic resonance imaging (MRI) technology. Additionally, this model could find application in various industrial sectors such as microfluidic devices, heat exchangers, the oil and gas industry, and chemical processing^1–3^. Several studies^4–10^ have been pursued to gain insights into blood flow in arteries under different assumptions. Chakravarty and Mandal^11^ investigated two-dimensional blood flow in tapered stenotic arteries, while Pontrelli^12^ focused on treating axisymmetric stenosis in arterial blood flow. Misra et al.^13^ conducted a rigorous study on blood flow through arteries with multiple stenoses. In recent decades, pulsatile blood flow has attracted substantial attention from researchers due to its crucial implications for understanding cardiovascular dynamics and associated pathologies. As a result, numerous investigations have explored the study of pulsatile blood flow, considering it a Newtonian fluid^14–16^. El-Shahed^17^ studied the pulsatile flow of blood in a stenosed porous medium subjected to periodic body acceleration. Additionally, Shit and Roy^18^ investigated the pulsatile blood flow within a constricted porous channel subjected to an external magnetic field. Notably, their findings revealed a crucial correlation between magnetic field intensity, blood flow reduction, and increasing Reynolds number. Rathod and Ravi^19^ focused on blood flow in stenosed inclined tubes with periodic body acceleration with a magnetic field. Jamil et al.^20^ explored the control of blood flow through stenosed porous arteries with a magnetic field. Their results highlighted the significant influence of the magnetic field on flow dynamics, unveiling practical applications for magnetic field therapy in treating cardiovascular diseases. Furthermore, Liu and Liu^21^ analyzed blood flow in tapered stenosed arteries under the influence of heat and mass transfer. Amos et al.^22^ investigated magnetohydrodynamic pulsatile blood flow in an inclined stenosed artery with body acceleration and slip effects. Their results illuminate the intricate relationships between magnetic field intensity and crucial flow parameters, including velocity, acceleration, shear stress, and volumetric flow rate. Manchi and Ponalagusamy^23^ investigated the pulsatile flow of an electromagnetic-hydrodynamic micropolar hybrid nanofluid within a porous bifurcated artery containing an overlapping stenosis. Ratchagar and Subasri^24^ explored the impact of Hall current on pulsatile blood flow within porous arteries with multiple stenoses. Considering the effects of slip velocity, their study identified regions characterized by low velocity and shear stress, providing valuable insights for understanding blood flow patterns in pathological conditions and during surgical interventions. Reddy et al.^25^ investigated the pulsatile hybrid nanofluid flow through a vertically permeable irregular channel with the impact of externally applied body acceleration. This study holds significant promise for various biomechanical applications, including radiation therapy for lung cancer treatment and regulating blood flow using magnetic fields during surgery.

Blood, a suspension of red cells containing haemoglobin with iron oxide, demonstrates electrical conductivity and magnetohydrodynamic flow properties. When an electrically conducting fluid is put into motion within a magnetic field, it creates electric and magnetic fields, generating a Lorentz force, which is a body force that obstructs fluid movement. This analysis is pertinent in various applications, including blood pumping, magnetic resonance imaging (MRI), and blood flow control during surgery. Numerous authors^26–33^ have delved into blood flow in arteries under the influence of magnetic fields in different scenarios. Gold^34^ provided an analytical solution for the magnetohydrodynamic equations, encompassing axial velocity and an axial-induced magnetic field. Misra and Shit^35^ investigated the behaviour of a viscoelastic electrically conducting fluid in a magnetic field, observing that higher magnetic field strength corresponds to increased blood temperature, suggesting new possibilities for heating methods. In separate work, Misra et al.^36^ conducted a mathematical study of single-phase stenosed arterial blood flow, investigating the complex flow behaviour under the influence of an applied magnetic field. Their research uncovered that changes in the intensity of the applied magnetic field can impact the wall shear stress in stenosed arteries, potentially leading to ruptures and subsequent paralysis in affected areas of the body. Ponalagusamy and Selvi^37^ presented a mathematical model for narrow arteries to examine the influence of external magnetic fields on two-phase blood flow, which consists of a central core of suspended erythrocytes and a surrounding cell-free layer. Their findings revealed reduced velocity profiles in the core and plasma regions as the magnetic field intensity increased.

Incorporating a porous medium into the study of fluid flow enriches its physical realism, particularly in modelling blood vessels and pulmonary systems where fatty deposits and artery blockages are present. Notable advancements in this field include the research conducted by Sorek and Sideman^38^, who examined blood flow in cardiac vessels using the Darcy-Forchheimer model, the investigation by Vankan et al.^39^ into non-Darcy transport in blood-perfused tissue, and the exploration of mass exchange employing an extended Darcy model by Preziosi and Farina^40^. Furthermore, Khaled and Vafai^41^ conducted a comprehensive review of heat and fluid dynamics applications within porous (biological) media. Ogulu and Amos^42^ investigated the impact of temporally varying wall mass flux on hydromagnetic pulsatile Newtonian blood flow within a Darcian porous cardiovascular system model using a regular perturbation technique. Additionally, Bhargava et al.^43^ analyzed pulsating magnetohydrodynamic blood flow and species diffusion within a porous medium channel by employing the Darcy-Forchheimer model. Reddy et al.^44^ investigated the entropy generation and heat transfer characteristics of a magnetohydrodynamic (MHD) silver-copper/blood hybrid nanofluid flowing over a porous plate. The study emphasizes the importance of understanding energy loss in biological systems and its potential applications in biomedical engineering and healthcare.

Heat transfer within the human body involves complex processes, including heat conduction within tissues, heat exchange due to the flow of arterial-venous blood through tissue pores (blood convection), metabolic heat production, and outside factors such as electromagnetic radiation emitted by electronic devices such as cell phones. These combined phenomena fall under bioheat transfer, a critical biomedical engineering area focused on understanding human body heat dynamics. Baish^45^ significantly contributed to this area by studying heat transport in counter-current blood vessels amidst arbitrary pressure gradients. Shrivastava et al.^46^ also presented an analytical investigation of heat transfer through finite tissue characterized by two blood vessels and uniform Dirichlet boundary conditions. The thermal conductivity of fluids can be significantly improved under specific conditions, especially using nanofluids containing nanoparticles. In recent years, nanoparticles have become widely recognized as versatile carriers for drugs, enabling targeted drug delivery while minimizing harm to non-target cells, particularly in the field of cancer treatment. Their remarkable adsorption capabilities make them highly beneficial in clinical applications for transporting drugs, proteins, and other substances to specific cellular targets. Previous studies^47–54^ have thoroughly examined the impact of nanoparticles in various scenarios. Ellahi et al.^55^ explored mixed convection nanofluid flow over a wedge, considering particle shape effects. Their findings highlight that increased volume friction and smaller particle size enhance heat transfer rates. Akbarzadeh et al.^56^ examined the flow of nanofluids under laminar conditions with forced convection in wavy channels, emphasizing the increased sensitivity of the average Nusselt number to the Reynolds number and channel aspect ratio as the aspect ratio grows. Sheikholeslami and Ganji^57^ investigated the impact of magnetic field on nanofluid flow between parallel plates. They found that skin friction coefficients rise with higher Squeeze and Hartman number but decrease as the nanofluid volume fraction increases. Rashidi et al.^58^ investigated heat transfer in nanofluid flow over a stretching sheet in the presence of a transverse magnetic field, thermal radiation, and buoyancy effects. Their study revealed that higher buoyancy parameters increase velocity profiles but decrease nanofluid temperature profiles. In a related study, Ellahi et al.^59^ examined the behaviour of nanoparticles in blood flow along permeable walls within stenosed arteries. Additionally, Sharma et al.^60^ developed a mathematical model to study the trajectories of magnetic nanoparticles within blood vessels under the influence of magnetic fields, with potential applications in magnetic drug targeting. Their research also demonstrated the deceleration of particles with magnetic forces. Nadeem and Ijaz^61^ investigated the influence of nanoparticles on the flow of blood in narrowed catheterized arteries, while Aman et al.^62^ examined the impact of gold nanoparticles on magnetohydrodynamic (MHD) Poiseuille flow of nanofluids in porous media, utilizing perturbation technique to solve the governing equations of the model. Reddy et al.^63^ investigated the flow of a gold-blood nanofluid through a microchannel driven by an electrokinetic force and analyses the associated entropy generation. The study highlights potential applications in understanding energy loss in biological systems and targeted cancer treatment.

In addressing the prevailing research landscape, characterized by a predominant focus on analysing momentum and heat transfer in one-dimensional models with a static pressure gradient, simplifying it into steady and unsteady components that remained constant with the length of the artery, our study presents a novel approach to fill existing gaps. This work aims to investigate the behaviour of unsteady pulsatile flow in a two-dimensional sinusoidal wavy channel. This departure from the conventional one-dimensional approach is motivated by the need to comprehensively understand and address the limitations observed in current literature. Building upon the findings of Chow and Abumandour et al.^64,65^, who highlighted significant pressure gradient variations, particularly in restricted channel length. This analysis considers the variations in pressure gradient, including the impact of magnetic field, nanoparticle volume fraction, radiation, and heat source parameters. The governing equations of nano-blood flow in a horizontal wavy channel are solved by the perturbation technique. The analytical solutions of stream function, velocity, wall shear stress, pressure gradient, and temperature are illustrated graphically, considering various values of the pertinent physical parameters.

## Mathematical formulation

**Figure 1:**
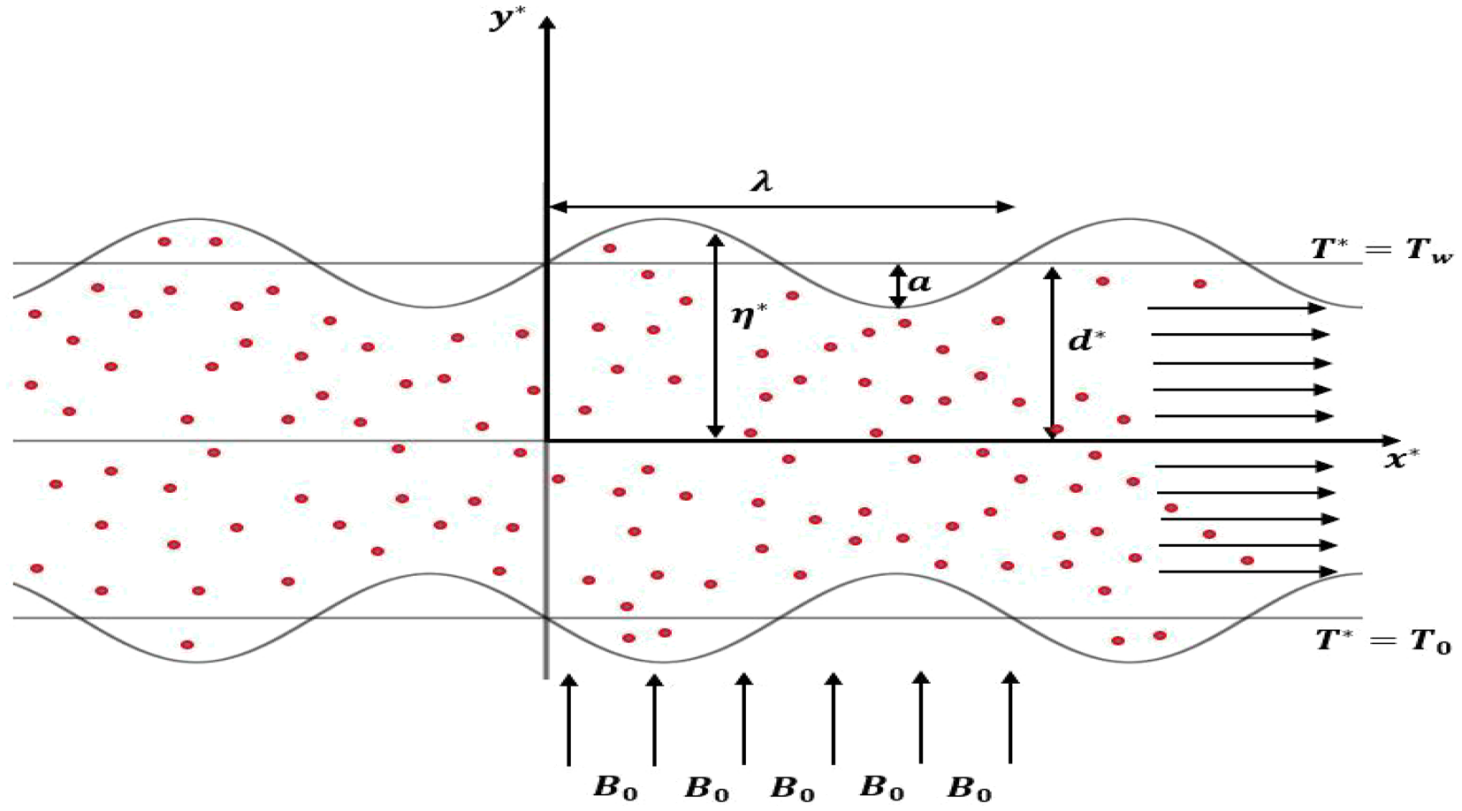
A schematic diagram for the flow geometry.

The research investigates the unsteady, incompressible flow of a Newtonian nanofluid through a symmetric, two-dimensional porous sinusoidal-wall channel, as depicted in Fig. 1^16^. This study aims to analyse the behaviour of nano-blood flow with a magnetic field and heat transfer. Specifically, a uniform magnetic field ${B}_{0}$ is applied to the pulsatile nano-blood flow in the transverse direction. Also, the temperature of the bottom wall is denoted by ${T}_{0}$, while ${T}_{w}$ indicates the temperature of the top wall. The boundary of the channel wavy walls is expressed by:

1 $${\eta }^{*}={d}^{*}+a sin\left(\frac{2\pi }{\lambda }{x}^{*}\right).$$

where ${x}^{*}$ is the longitudinal axis of the channel, $a$ is the height of the wall constriction, ${d}^{*}$ is the half width of the channel, $\lambda$ is the length of wall constriction. Under the above considerations, the governing equations for conservative momentum and energy in general form are expressed as follow^33,54,56^:

A. Continuity equation,

2 $$\frac{\partial {{\text{u}}}^{*}}{\partial {{\text{x}}}^{*}}+\frac{\partial {{\text{v}}}^{*}}{\partial {{\text{y}}}^{*}}=0,$$

B. Momentum equation,

3 $$\frac{\partial {{\text{u}}}^{*}}{\partial {{\text{t}}}^{*}}+{{\text{u}}}^{*}\frac{\partial {{\text{u}}}^{*}}{\partial {{\text{x}}}^{*}}+{{\text{v}}}^{*}\frac{\partial {{\text{u}}}^{*}}{\partial {{\text{y}}}^{*}}=-\frac{1}{{\uprho }_{{\text{nf}}}}\frac{\partial {{\text{p}}}^{*}}{\partial {{\text{x}}}^{*}}+ {\upupsilon }_{{\text{nf}}}\left(\frac{{\partial }^{2}{{\text{u}}}^{*}}{\partial {{{\text{x}}}^{*}}^{2}}+\frac{{\partial }^{2}{{\text{u}}}^{*}}{\partial {{{\text{y}}}^{*}}^{2}}\right)-\left(\frac{\upsigma {{\text{B}}}_{{\text{O}}}^{2}}{{\uprho }_{{\text{nf}}}}\right){{\text{u}}}^{*}-\left(\frac{{\upupsilon }_{{\text{nf}}}}{{\text{k}}}\right){{\text{u}}}^{*},$$

4 $$\frac{\partial {{\text{v}}}^{*}}{\partial {{\text{t}}}^{*}}+{{\text{u}}}^{*}\frac{\partial {{\text{v}}}^{*}}{\partial {{\text{x}}}^{*}}+{{\text{v}}}^{*}\frac{\partial {{\text{v}}}^{*}}{\partial {{\text{y}}}^{*}}=-\frac{1}{{\uprho }_{{\text{nf}}}}\frac{\partial {{\text{p}}}^{*}}{\partial {{\text{y}}}^{*}}+ {\upupsilon }_{{\text{nf}}}\left(\frac{{\partial }^{2}{{\text{v}}}^{*}}{\partial {{{\text{x}}}^{*}}^{2}}+\frac{{\partial }^{2}{{\text{v}}}^{*}}{\partial {{{\text{y}}}^{*}}^{2}}\right),$$

C. Energy equation,

5 $$\frac{\partial {{\text{T}}}^{*}}{\partial {{\text{t}}}^{*}}+{{\text{u}}}^{*}\frac{\partial {{\text{T}}}^{*}}{\partial {{\text{x}}}^{*}}+{{\text{v}}}^{*}\frac{\partial {{\text{T}}}^{*}}{\partial {{\text{y}}}^{*}}=\frac{{{\text{k}}}_{{\text{nf}}}}{{\left(\uprho {{\text{C}}}_{{\text{p}}}\right)}_{{\text{nf}}}}\left(\frac{{\partial }^{2}{{\text{T}}}^{*}}{\partial {{{\text{y}}}^{*}}^{2}}\right)-\frac{1}{{\left(\uprho {{\text{C}}}_{{\text{p}}}\right)}_{{\text{nf}}}}\frac{\partial {{\text{q}}}_{{\text{r}}}}{\partial {{\text{y}}}^{*}}+\frac{{{\text{Q}}}_{0}}{{\left(\uprho {{\text{C}}}_{{\text{p}}}\right)}_{{\text{nf}}}}\left({{\text{T}}}^{*}-{{\text{T}}}_{0}\right).$$

Rosseland approximation for radiative heat flux, ${{\text{q}}}_{{\text{r}}}$ is defined as^66^:

6 $${{\text{q}}}_{{\text{r}}}=-\left(\frac{4{\upsigma }^{*}}{3{{\text{k}}}^{*}}\frac{\partial {{\text{T}}}^{*4}}{\partial {{\text{y}}}^{*}}\right).$$

The Rosseland mean absorption coefficient, denoted as ${k}^{*}$, and the Stefan–Boltzmann constant, denoted as ${\sigma }^{*}$. It is assumed that the variation of the temperature within the flow is small enough to permit the expansion of ${T}^{*4}$ in a Taylor’s series. The expansion of ${T}^{*4}$ around ${T}_{0}$ and the neglect of higher-order terms result in the following expression^67^:

7 $${T}^{*4}\cong 4{T}_{0}^{3}{T}^{*}-3{T}_{0}^{4}.$$

Upon substituting Eqs. (6, 7) into Eq. (5), the following expression is obtained:

8 $$\frac{\partial {T}^{*}}{\partial {t}^{*}}+{u}^{*}\frac{\partial {T}^{*}}{\partial {x}^{*}}+{v}^{*}\frac{\partial {T}^{*}}{\partial {y}^{*}}=\frac{{k}_{nf}}{{\left(\rho {C}_{p}\right)}_{nf}}\left(\frac{{\partial }^{2}{T}^{*}}{\partial {{y}^{*}}^{2}}\right)+\frac{16{\sigma }^{*}{T}_{0}^{3}}{{3{k}^{*}\left(\rho {C}_{p}\right)}_{nf}}\left(\frac{{\partial }^{2}{T}^{*}}{\partial {{\text{y}}}^{*2}}\right)+\frac{{Q}_{0}}{{\left(\rho {C}_{p}\right)}_{nf}}\left({T}^{*}-{T}_{0}\right).$$

The wall boundary conditions for the porous channel under the no-slip condition can be expressed in the following manner^16,54^:

9a $$\left.\begin{array}{c}{u}^{*}=0 \\ \\ {v}^{*}=0 \end{array} \right\} \, at \, {y}^{*}=\pm {\eta }^{*},$$

9b $${T}^{*}={T}_{w} at \, {y}^{*}={\eta }^{*},$$

9c $${T}^{*}={T}_{0} at \, {y}^{*}=-{\eta }^{*}.$$

where ${u}^{*},{v}^{*}$ are the velocity components of the nanofluid in ${x}^{*},{y}^{*}$ directions respectively, ${p}^{*}$ is the nanofluid pressure, ${\rho }_{nf}$, ${\upsilon }_{nf}$ are the nanofluid density and kinematic viscosity respectively, $\sigma$ is the electrical conductivity, ${B}_{0}$ is the uniform magnetic field, ${T}^{*}$ is the temperature of nanofluid, ${\left(\rho {C}_{p}\right)}_{nf}$ is the specific heat capacity of the nanofluid, ${k}_{nf}$ is the thermal conductivity of the nanofluid, ${q}_{r}$ is the radiative heat flux, ${Q}_{0}$ is the heat source/sink parameter, and ${t}^{*}$ refers to the time.

The thermophysical properties of the nanofluid, as presented by Zahir et al.^68^, are as follows:

10 $$\left.\begin{array}{c}\begin{array}{c}\begin{array}{c}\begin{array}{c}{\rho }_{nf}=\left(1-\varphi \right){\rho }_{f}+\varphi {\rho }_{n}, \\ {\mu }_{nf}=\frac{{\mu }_{f}}{(1-\varphi {)}^{2.5}}, \end{array}\\ {\upsilon }_{nf}=\frac{{\mu }_{nf}}{{\rho }_{nf}} , \end{array}\\ {\left(\rho {C}_{p}\right)}_{nf}=\left(1-\varphi \right){\left(\rho {C}_{p}\right)}_{f}+\varphi {\left(\rho {C}_{p}\right)}_{n},\end{array}\\ \frac{{k}_{nf}}{{k}_{f}}=\frac{\left(2{k}_{f}+{k}_{n}\right)-2\varphi \left({k}_{f}-{k}_{n}\right)}{\left(2{k}_{f}+{k}_{n}\right)+\varphi \left({k}_{f}-{k}_{n}\right)}.\end{array}\right\}$$

Furthermore, the function ${\psi }^{*}$ is chosen in the following manner:

11 $$\left.\begin{array}{c}{u}^{*}= \frac{\partial {\psi }^{*}}{\partial {y}^{*}} , \\ {v}^{*}=-\frac{\partial {\psi }^{*}}{\partial {x}^{*}}.\end{array}\right\}$$

After substituting ${\psi }^{*}$ into Eqs. (3–4), and (8), and eliminating the pressure from Eqs. (3) and (4), these equations can be expressed as follows:

12 $$\frac{\partial }{\partial {t}^{*}}\left({\nabla }^{2}{\psi }^{*}\right)+\frac{\partial {\psi }^{*}}{\partial {y}^{*}}{\nabla }^{2}\frac{\partial {\psi }^{*}}{\partial {x}^{*}}-\frac{\partial {\psi }^{*}}{\partial {x}^{*}}{\nabla }^{2}\frac{\partial {\psi }^{*}}{\partial {y}^{*}}={\upsilon }_{nf}{\nabla }^{4}{\psi }^{*}-\left(\frac{\sigma {B}_{O}^{2}}{{\rho }_{nf}}+\frac{{\upsilon }_{nf}}{k}\right)\frac{{\partial }^{2}{\psi }^{*}}{\partial {{y}^{*}}^{2}},$$

13 $$\frac{\partial {T}^{*}}{\partial {t}^{*}}+\frac{\partial {\psi }^{*}}{\partial {y}^{*}}\frac{\partial {T}^{*}}{\partial {x}^{*}}-\frac{\partial {\psi }^{*}}{\partial {x}^{*}}\frac{\partial {T}^{*}}{\partial {y}^{*}}=\frac{{k}_{nf}}{{\left(\rho {C}_{p}\right)}_{nf}}\left(\frac{{\partial }^{2}{T}^{*}}{\partial {{y}^{*}}^{2}}\right)+\frac{16{\sigma }^{*}{T}_{0}^{3}}{{3{k}^{*}\left(\rho {C}_{p}\right)}_{nf}}\left(\frac{{\partial }^{2}{T}^{*}}{\partial {{\text{y}}}^{*2}}\right)+\frac{{Q}_{0}}{{\left(\rho {C}_{p}\right)}_{nf}}\left({T}^{*}-{T}_{0}\right).$$

where

14 $${\nabla }^{2}=\frac{{\partial }^{2}}{\partial {{x}^{*}}^{2}}+\frac{{\partial }^{2}}{\partial {{y}^{*}}^{2}},$$

The relevant boundary conditions are as follows:

15a $$\left.\begin{array}{c}\frac{\partial {\psi }^{*}}{\partial {y}^{*}}=0\\ \frac{\partial {\psi }^{*}}{\partial {x}^{*}}=0\end{array} \right\} \, at \, {y}^{*}=\pm {\eta }^{*},$$

15b $${\psi }^{*}=0 \;at \;{y}^{*}=0,$$

15c $${\psi }^{*}=Q \;\left(constant\right) \;at \;{y}^{*}={\eta }^{*},$$

15d $${T}^{*}={T}_{w} \;at \, \;{y}^{*}={\eta }^{*},$$

15e $${T}^{*}={T}_{0} \;at \, \;{y}^{*}=-{\eta }^{*}.$$

Introducing the following non-dimensional variables as follows:

16 $$\left.\begin{array}{c}\begin{array}{c}x=\frac{{x}^{*}}{\lambda }, y=\frac{{y}^{*}}{d}, \eta =\frac{{\eta }^{*}}{d}, \\ \zeta =\frac{y}{\eta }, t=\frac{{{\upsilon }_{f} t}^{*}}{\lambda d}, \psi =\frac{{\psi }^{*}}{{\upsilon }_{f}}, \end{array}\\ P=\frac{\lambda d{P}^{*}}{{\rho }_{f}{\upsilon }_{f}^{2}}, \theta =\frac{{T}^{*}-{T}_{0}}{{T}_{w}-{T}_{0}}.\end{array}\right\}$$

Using the dimensionless variables stated above, Eqs. (12–15) are obtained as follows:

17 $${\nabla }^{4}\psi -\left(H{a}^{2}(1-\varphi {)}^{2.5}+\frac{1}{Da}\right)\frac{{\partial }^{2}\psi }{\partial {y}^{2}}=\delta (1-\varphi {)}^{2.5}\left(1-\varphi +\left(\frac{\varphi {\rho }_{n}}{{\rho }_{f}} \right)\right)\left(\frac{\partial }{\partial t}\left({\nabla }^{2}\psi \right)+\frac{\partial \psi }{\partial y}{\nabla }^{2}\frac{\partial \psi }{\partial x}-\frac{\partial \psi }{\partial x}{\nabla }^{2}\frac{\partial \psi }{\partial y}\right),$$

18 $$\left(\frac{\left(\left(\frac{{k}_{nf}}{{k}_{f}}\right)+\left(\frac{4}{3}\right)Rd\right)}{Pr}\frac{{\partial }^{2}\theta }{\partial {y}^{2}}+{Q}_{t}\theta \right)=\delta \left(1-\varphi +\left(\frac{\varphi {\left(\rho {C}_{p}\right)}_{n}}{{\left(\rho {C}_{p}\right)}_{f}} \right)\right)\left(\frac{\partial \theta }{\partial t}+\frac{\partial \psi }{\partial y}\frac{\partial \theta }{\partial x}-\frac{\partial \psi }{\partial x}\frac{\partial \theta }{\partial y}\right).$$

where the amplitude ratio $\varepsilon$, the wall slope parameter $\delta$, Hartmann number $Ha$, Darcy number $Da$ , Prandtl number $Pr$, the radiation parameter $Rd$, and the heat source parameter ${Q}_{t}$ are defined respectively by:

19 $$\left.\begin{array}{c}\varepsilon =\frac{a}{d}, \delta =\frac{d}{\lambda }, \\ Ha={B}_{0}d\sqrt{\frac{\sigma }{{\mu }_{f}}, } Da=\frac{k}{{d}^{2}}, \\ Pr=\frac{{\left(\mu {C}_{p}\right)}_{f}}{{k}_{f}}, Rd=\frac{4{\sigma }^{*}{T}_{0}^{3}}{{k}_{f}{k}^{*}}, \\ { Q}_{t}=\frac{{Q}_{0}{d}^{2}}{{\left(\rho {C}_{p}\right)}_{f}{\nu }_{f}}, {\nabla }^{2}={\delta }^{2}\frac{{\partial }^{2}}{\partial {{x}^{*}}^{2}}+\frac{{\partial }^{2}}{\partial {{y}^{*}}^{2}}. \\ \end{array}\right\}$$

The boundary conditions corresponding to this transformation are as follows:

20a $$\left.\begin{array}{c}\frac{\partial \psi }{\partial y}=0\\ \frac{\partial \psi }{\partial x}=0 \end{array} \right\} \, at \, y=\pm \eta ,$$

20b $$\psi =0 \;at \;y=0,$$

20c $$\psi =Q \;\left(constant\right) \;at \;y=\eta ,$$

20d $$\theta =1 \;at \;y=\eta ,$$

20e $$\theta =0 \;at \;y=-\eta .$$

### Solution method

After applying the dimensionless technique, it is possible to assume that the stream function $\psi$, temperature $\theta$, and pressure $P$ have expansions in terms of the small parameter δ, representing the channel slope, as indicated in Ref.^16^, these expansions can be expressed as follows:

21a $$\psi ={\psi }_{0}+\delta {\psi }_{1}+{\delta }^{2}{\psi }_{2}+...,$$

21b $$\theta ={\theta }_{0}+\delta {\theta }_{1}+{\delta }^{2}{\theta }_{2}+...,$$

21c $$P={P}_{0}+\delta {P}_{1}+{\delta }^{2}{P}_{2}+...,$$

By substituting Eq. (21a, 21b, 21c) into Eqs. (17–20) and collecting terms of the same powers of δ, including zero and first order terms, yields the subsequent perturbed equations:

Zero order:

22 $$\frac{{\partial }^{4}{\psi }_{0} }{\partial {y}^{4}}-{m}^{2}\frac{{\partial }^{2}{\psi }_{0} }{\partial {y}^{2}}= 0,$$

23 $$\frac{{\partial }^{2}{\theta }_{0} }{\partial {y}^{2}}+{m}_{1}^{2}{\theta }_{0}=0$$

24a $$\left.\begin{array}{c}\frac{\partial {\psi }_{0}}{\partial y}=0\\ \frac{\partial {\psi }_{0}}{\partial x}=0 \end{array} \right\} \, at \, y=\pm \eta ,$$

24b $${\psi }_{0}=Q \;\left(constant\right) \;at \;y=\eta ,$$

24c $${\psi }_{0}=0 \;at \;y=0,$$

24d $${\theta }_{0}=1 \;at \;y=\eta ,$$

24e $${\theta }_{0}=0 \;at \;y=-\eta .$$

First order:

25 $$\frac{{\partial }^{4}{\psi }_{1} }{\partial {y}^{4}}-{m}^{2}\frac{{\partial }^{2}{\psi }_{1} }{\partial {y}^{2}}={B}_{1}\left(\frac{{\partial }^{3}{\psi }_{0}}{\partial t\partial {y}^{2}}+\frac{\partial {\psi }_{0}}{\partial y}\frac{{\partial }^{3}{\psi }_{0}}{\partial x\partial {y}^{2}}-\frac{\partial {\psi }_{0}}{\partial x}\frac{{\partial }^{3}{\psi }_{0}}{\partial {y}^{3}}\right),$$

26 $$\frac{{\partial }^{2}{\theta }_{1} }{\partial {y}^{2}}+{m}_{1}^{2}{\theta }_{1}={B}_{2}\left(\frac{\partial {\theta }_{0}}{\partial t}+\frac{\partial {\psi }_{0}}{\partial y}\frac{\partial {\theta }_{0}}{\partial x}-\frac{\partial {\psi }_{0}}{\partial x}\frac{\partial {\theta }_{0}}{\partial y}\right),$$

27a $$\left.\begin{array}{c}\frac{\partial {\psi }_{1}}{\partial y}=0\\ \frac{\partial {\psi }_{1}}{\partial x}=0\end{array} \right\} \, at \, y=\pm \eta ,$$

27b $${\psi }_{1}=0 \;at \;y=\eta ,$$

27c $${\psi }_{1}=0 \;at \;y=0,$$

27d $${\theta }_{1}=0 \;at \;y=\eta ,$$

27e $${\theta }_{1}=0 \;at \;y=-\eta .$$

Furthermore, it is assumed:

28a $${\psi }_{0}={\psi }_{00}\left(x,y\right){e}^{i\omega t},$$

28b $${\theta }_{0}={\theta }_{00}\left(x,y\right){e}^{i\omega t},$$

28c $${P}_{0}={P}_{00}\left(x,y\right){e}^{i\omega t},$$

28d $${\psi }_{1}={\psi }_{10}\left(x,y\right)+{\psi }_{11}\left(x,y\right){e}^{i\omega t}+{\psi }_{12}\left(x,y\right){e}^{2i\omega t},$$

28e $${\theta }_{1}={\theta }_{10}\left(x,y\right)+{\theta }_{11}\left(x,y\right){e}^{i\omega t}+{\theta }_{12}\left(x,y\right){e}^{2i\omega t},$$

28f $${P}_{1}={P}_{10}\left(x,y\right)+{P}_{11}\left(x,y\right){e}^{i\omega t}+{P}_{12}\left(x,y\right){e}^{2i\omega t}.$$

By substituting Eqs. (28a, 28b, 28c, 28d, 28e, 28f) into Eqs. (22–27), equating similar harmonic terms, and solving the resulting partial differential equations under the corresponding boundary conditions, the following results are obtained:

29 $$\psi =Q{C}_{1}m \left(\frac{{\text{sinh}}\left(my\right)}{m}-y {\text{cosh}}\left(m\eta \right) \right){e}^{i\omega t}+\delta \left\{{C}_{2}\left[\frac{{C}_{1}{C}_{3}}{2}\left(y-\frac{\eta {\text{sinh}}\left(my\right)}{{\text{sinh}}\left(m\eta \right)}\right)-\frac{\eta {\text{cosh}}\left(\mathit{m\eta }\right){\text{sinh}}\left(my\right)}{2{m}^{3}{\text{sinh}}\left(m\eta \right)}+\frac{y{\text{cosh}}\left(my\right)}{2{m}^{3}}\right]{e}^{i\omega t}+{C}_{4}\left[{\text{sinh}}\left(m\eta \right)\left(\frac{2\mathrm{ sinh}\left(my\right)}{{m}^{5}}-\frac{5 y{\text{cosh}}\left(my\right)}{4{m}^{4}}+\frac{{y}^{2} {\text{sinh}}\left(my\right)}{4{m}^{3}}\right)-\eta {\text{cosh}}\left(m\eta \right)\left(\frac{ y{\text{cosh}}\left(my\right)}{2{m}^{3}}-\frac{\mathrm{ sinh}\left(my\right)}{{m}^{4}}\right)+{C}_{5}\left(\frac{{\text{sinh}}\left(my\right)}{{m}^{2}}-\frac{y {\text{sinh}}\left(m\eta \right) }{\eta {m}^{2}}\right)+{C}_{6}y\right]{e}^{2i\omega t}\right\},$$

30 $$\theta =\left[\frac{{\text{cos}}\left({m}_{1}y\right)}{2{\text{cos}}\left({m}_{1}\eta \right)}+\frac{{\text{sin}}\left({m}_{1}y\right)}{2{\text{sin}}\left({m}_{1}\eta \right)}\right]+\delta \left\{{C}_{7}\left[{C}_{8}{\text{cos}}\left({m}_{1}y\right)+{C}_{9}{\text{sin}}\left({m}_{1}y\right)+{C}_{10} \frac{{\text{sin}}\left({m}_{1}y\right){\text{sinh}}\left(my\right)}{m({m}^{2}+4{m}_{1}^{2})}+{C}_{11} \frac{{\text{cos}}\left({m}_{1}y\right){\text{cosh}}\left(my\right)}{m({m}^{2}+4{m}_{1}^{2})}+{C}_{12} \frac{{\text{cos}}\left({m}_{1}y\right){\text{sinh}}\left(my\right)}{m({m}^{2}+4{m}_{1}^{2})}+{C}_{13} \frac{{\text{sin}}\left({m}_{1}y\right){\text{cosh}}\left(my\right)}{m({m}^{2}+4{m}_{1}^{2})}+{C}_{14}\frac{y{\text{sin}}\left({m}_{1}y\right)}{4{m}_{1}^{2}}+{C}_{15}\frac{y{\text{cos}}\left({m}_{1}y\right)}{4{m}_{1}^{2}}+{C}_{16}\frac{{y}^{2}{\text{sin}}\left({m}_{1}y\right)}{4{m}_{1}}+{C}_{17}\frac{{y}^{2}{\text{cos}}\left({m}_{1}y\right)}{4{m}_{1}}\right]{e}^{i\omega t}\right\},$$

The axial velocity can be determined by substituting Eq. (29) into (11):

31 $$u=Q{C}_{1}m {e}^{i\omega t}\left({\text{cosh}}\left(my\right)-{\text{cosh}}\left(m\eta \right) \right)+\delta \left\{{C}_{2}\left[\frac{{C}_{1}{C}_{3}}{2}\left(1-\frac{\eta m{\text{cosh}}\left(my\right)}{{\text{sinh}}\left(m\eta \right)}\right)-\left(\frac{\eta {\text{cosh}}\left(m\eta \right)}{2{m}^{2}{\text{sinh}}\left(m\eta \right)}-\frac{1}{2{m}^{3}}\right){\text{cosh}}\left(my\right)+\frac{y{\text{sinh}}\left(my\right)}{2{m}^{2}}\right]{e}^{i\omega t}+{C}_{4}\left[{\text{sinh}}\left(m\eta \right)\left(\frac{3{\text{cosh}}\left(my\right)}{4 {m}^{4}}-\frac{3 y{\text{sinh}}\left(my\right)}{4{m}^{3}}+\frac{{y}^{2} {\text{cosh}}\left(my\right)}{4{m}^{2}}\right)-\eta {\text{cosh}}\left(m\eta \right)\left(\frac{ y{\text{sinh}}\left(my\right)}{2{m}^{2}}-\frac{{\text{cosh}}\left(my\right)}{2{m}^{3}}\right)+{C}_{5}\left(\frac{{\text{cosh}}\left(my\right)}{m}-\frac{ {\text{sinh}}\left(m\eta \right) }{\eta {m}^{2}}\right)+{C}_{6}\right]{e}^{2i\omega t}\right\},$$

The non-dimensional shear stress exerted on the wall is expressed as:

32 $${\tau }_{w}={\left[\frac{{\partial }^{2}\psi }{\partial {y}^{2}}\right]}_{y=\eta },$$

By employing Eq. (29) in (32), the wall shear stress can be expressed as:

33 $${\tau }_{w}=Q{C}_{1}{m}^{2} {e}^{i\omega t} {\text{sinh}}\left(m\eta \right)+\delta \left\{{C}_{2}\left[\frac{{\text{sinh}}\left(m\eta \right)}{{m}^{2}}-\frac{{C}_{1}{C}_{3} \eta {m}^{2}}{2}\right]{e}^{i\omega t}+{C}_{4}\left[{\text{sinh}}\left(m\eta \right)\left(\frac{{\eta }^{2}{\text{sinh}}\left(m\eta \right)}{4m}-\frac{ \eta {\text{cosh}}\left(m\eta \right)}{4{m}^{2}}\right)-\frac{ {\eta }^{2}{{\text{cosh}}}^{2}\left(m\eta \right)}{2m}+{C}_{5} {\text{sinh}}\left(m\eta \right)\right]{e}^{2i\omega t}\right\},$$

The non-dimensional axial pressure gradient can be derived from Eq. (2) as follows:

34 $$P=\frac{\partial p/\partial x}{{\rho }_{f}{v}_{f}^{2}/{d}^{3}}=\left(\frac{1}{(1-\varphi {)}^{2.5}}\right)\left({\nabla }^{2}\frac{\partial \psi }{\partial y}-{m}^{2}\frac{\partial \psi }{\partial y}-\delta {B}_{1}\left(\frac{{\partial }^{2}\psi }{\partial t\partial y}+\frac{\partial \psi }{\partial y}\frac{{\partial }^{2}\psi }{\partial x\partial y}-\frac{\partial \psi }{\partial x}\frac{{\partial }^{2}\psi }{\partial {y}^{2}}\right)\right),$$

By substituting Eqs. (21a, 21b, 21c) and (28a, 28b, 28c, 28d, 28e, 28f) into Eq. (34), equating similar terms, and simplifying, results in:

35 $$P=\frac{Q{C}_{1}{m}^{3} {e}^{i\omega t}}{(1-\varphi {)}^{2.5}}{\text{cosh}}\left(m\eta \right) +\frac{\delta }{(1-\varphi {)}^{2.5}}\left\{{C}_{2}\left[\frac{{\text{cosh}}\left(m\eta \right)}{m}-\frac{{C}_{1}{C}_{3} {m}^{2}}{2}\right]{e}^{i\omega t}+{C}_{4}\left[\frac{-{\text{sinh}}\left(m\eta \right){\text{cosh}}\left(m\eta \right)}{{m}^{2}}-\left(\frac{\eta }{m}\right)+\frac{ {C}_{5}{\text{sinh}}\left(m\eta \right)}{\eta }-{C}_{6}{m}^{2}\right]{e}^{2i\omega t}\right\},$$

The coefficients ${C}_{1},{C}_{2},{C}_{3},$ · · · etc. are provided in the Appendix.

### Validation of results

**Figure 2:**
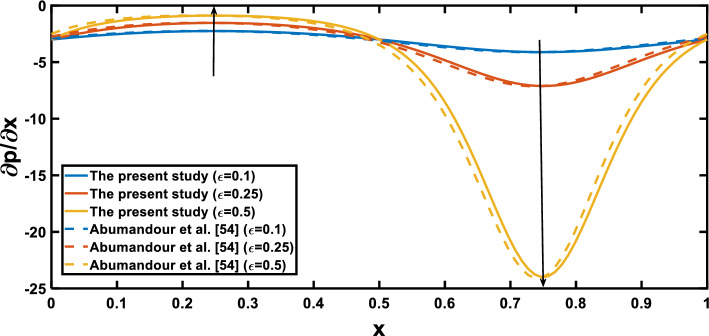
Pressure gradient for various values of the amplitude ratio $\left(\varepsilon \right)$.

For validation, the present results for the pulsatile flow of the base fluid (i.e. with $\varphi =0$) are compared with those obtained by Abumandour et al.^65^. In the case of steady flow, Fig. 2 shows a good agreement of the present results, specifically the variation of pressure gradient with axial distance^65^ for the amplitude ratio parameter $\epsilon =0.1, 0.25, 0.5$. with $Ha=\varphi =\frac{1}{Da}=Rd=Pr={Q}_{t}=0.$

## Results and discussion

**Figure 3:**
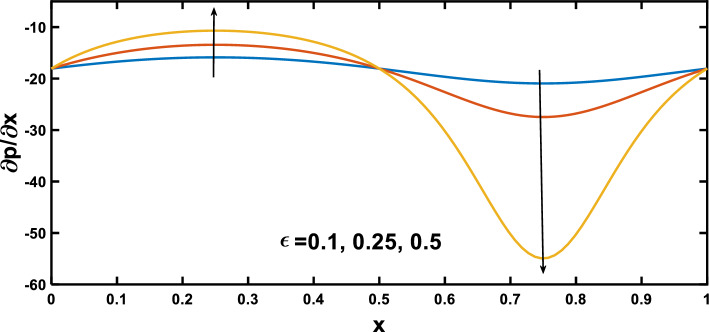
Pressure gradient for various values of the amplitude ratio $\left(\varepsilon \right)$.

In this section, numerical simulations were conducted to investigate the impact of biophysical parameters, including the amplitude ratio parameter, Hartmann number, Darcy number, nanoparticle concentration, radiation parameter, and Prandtl number, on profiles of pressure gradient, velocity, wall shear stress, and temperature, as governed by Eqs. (29–35). The graphical representation of these profiles can be observed in Figs. 3, 19. Table 1 provides the default values for the biophysical parameters utilized in the simulation.

**Table 1:** Default values for critical parameters employed in simulations.

Parameter	$\delta$	$t$	$\omega$	$\varepsilon$	$Da$	$Ha$	$\varphi$	$Pr$	$Rd$	${Q}_{t}$
Value	0.1	$2\pi$	1	0.25	0.1	2	0.1	14	0.2	1

Moreover, Table 2 presents the thermophysical numerical parameters for both blood and gold nanoparticles^69,70^.

**Table 2:** Numerical values of base fluid and nanoparticles.

Materials thermophysical properties	Base fluid (blood)	Nanoparticles (gold)
Density [$\rho (kg/{m}^{3})$]	1063	19,320
Heat capacitance [${c}_{p}(J/kgK )$]	3594	129
Thermal conductivity [$k(W/mK)$ ]	0.492	314

**Figure 4:**
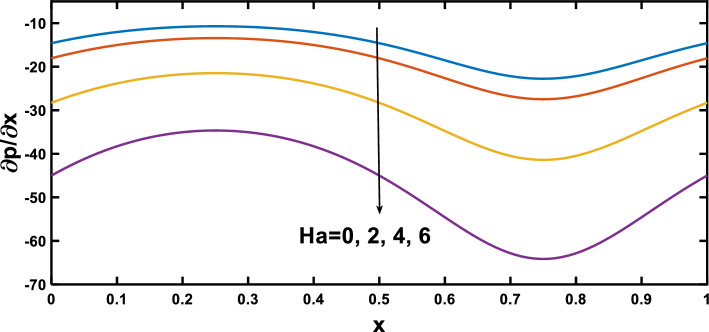
Pressure gradient for various values of Hartmann number $\left(Ha\right)$.

**Figure 5:**
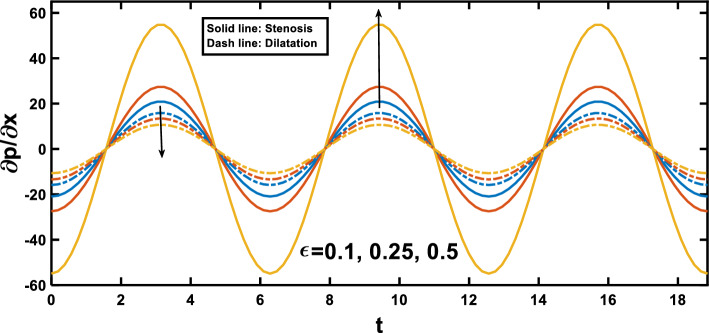
Pressure gradient for various values of the amplitude ratio $\left(\varepsilon \right)$ over time.

**Figure 6:**
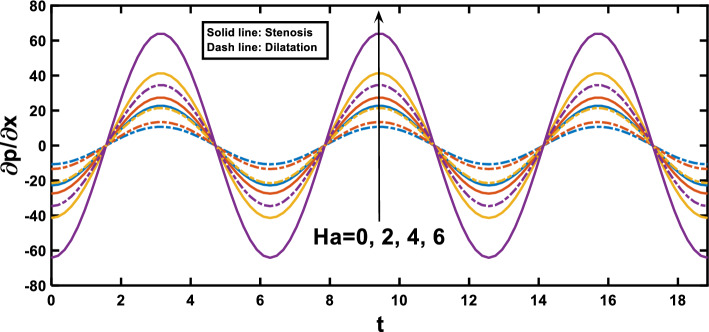
Pressure gradient for various values of Hartmann number $\left(Ha\right)$ over time.

Figures 3, 4 show the variation of the pressure gradient with axial distance. The interaction between the amplitude ratio parameter and the pressure gradient variation along the channel wall is depicted in Fig. 3, specifically in the context of stenosis and aneurysm. In regions with stenosis, an increased amplitude ratio parameter intensifies the pressure gradient. Conversely, a heightened amplitude ratio parameter in aneurysms diminishes the pressure gradient along the axial distance. Considering the boundary layer thickness, the pressure gradient variation across aneurysms is less pronounced than across stenotic regions. The dilation of aneurysms leads to a thicker boundary layer, resulting in lower shear stresses near the vessel wall. This decrease in shear stresses contributes to a lower pressure gradient across aneurysms, as opposed to the steeper pressure gradient across the stenosis, where the thinner boundary layer induces higher shear stresses and a more abrupt pressure gradient, and this observation agrees qualitatively well with^65^. Figure 4 depicts the variation of the pressure gradient along the length of the stenosis for different Hartmann number values. The pressure gradient rises with an increase in the Hartmann number, as demonstrated by^53^. This phenomenon can be attributed to the intensified effect of the magnetic field on the fluid flow at higher Hartmann number values. The Lorentz force exerted by the magnetic field acts as an additional resistance to the flow. Consequently, a higher pressure gradient is required to maintain the same flow rate through the channel. Figure 5 illustrates the periodic variation of the pressure gradient over time. In regions with stenosis, a rise in the amplitude ratio parameter leads to an increase in the peak value of each oscillation. Conversely, in the segments with an aneurysm, an opposite trend is apparent, with elevated amplitude ratio parameters causing a decline in the peak values of each oscillation. Figure 6 illustrates that the pressure gradient varies periodically with time, and the peak value of each oscillation increases with the rise of the Hartmann number.

**Figure 7:**
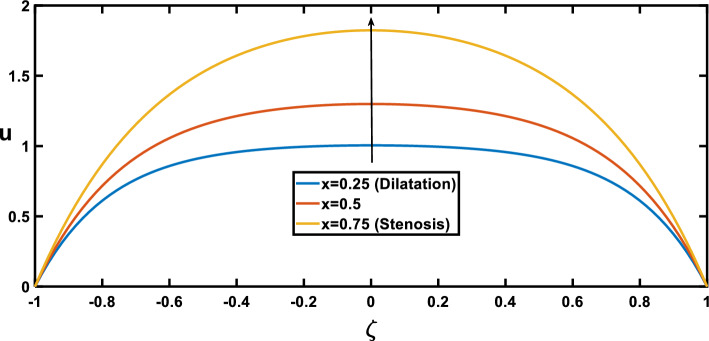
Velocity profile for various values of the cross sections $\left(x\right)$.

**Figure 8:**
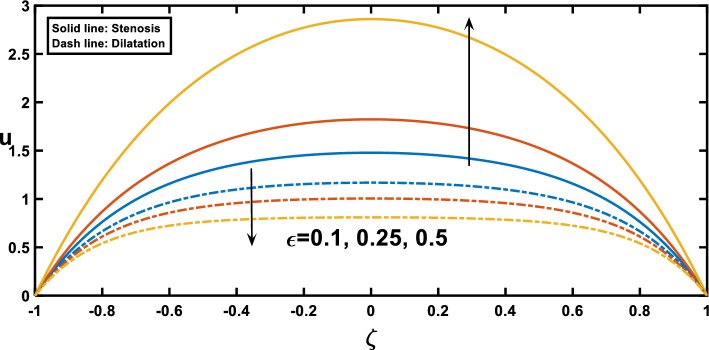
Velocity profile for various values of the amplitude ratio $\left(\varepsilon \right)$.

**Figure 9:**
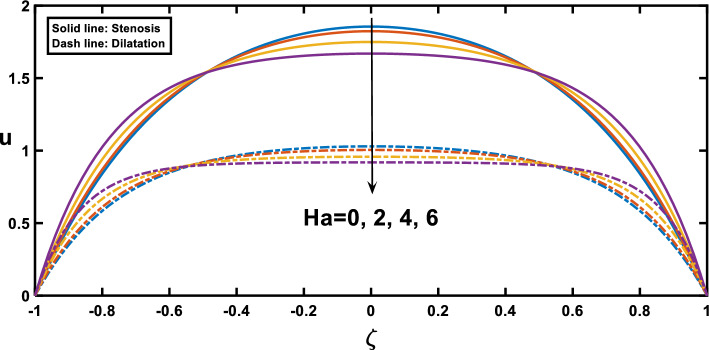
Velocity profile for various values of Hartmann number $\left(Ha\right)$.

**Figure 10:**
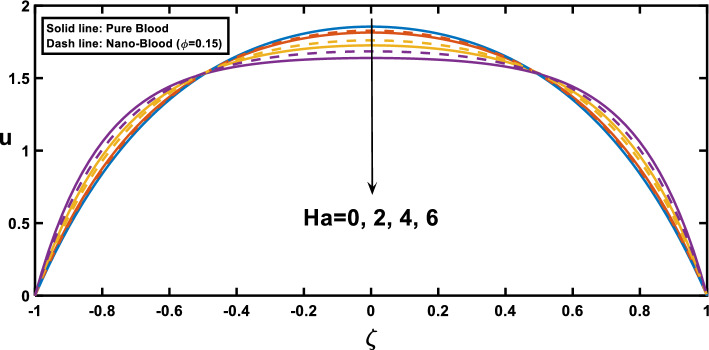
Velocity profile for various values of Hartmann number $\left(Ha\right)$ and the nanoparticle concentration $(\varphi )$.

**Figure 11:**
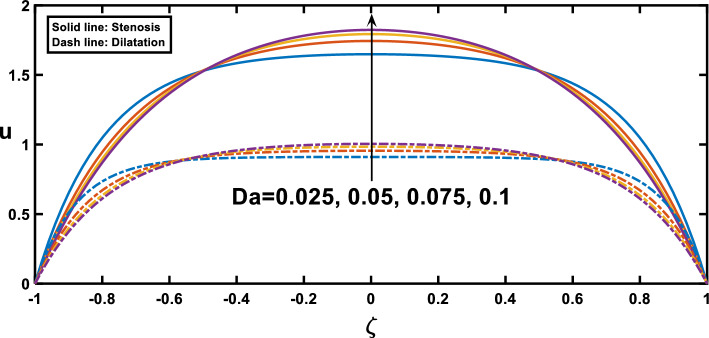
Velocity profile for various values of Darcy number $\left(Da\right)$.

The velocity profiles, depicted in Figs. 7, 8, 9, 10 and 11, offer insightful observations. Figure 7 shows the profile of velocity for the wavy channel geometry. As expected, velocity is maximum at the centre of the channel for x = 0.75 and minimum at x = 0.25, representing the stenosis and aneurysm segments, respectively. Building upon the findings of Figs. 3, 8 reveals a compelling trend in velocity variation with stenosis and aneurysm size. A notable velocity augmentation is observed with increasing stenosis size, while a reduction in velocity occurs with increasing aneurysm size. This aligns perfectly with the established literature^53^ and further reinforces the significance of the interplay between channel geometry and pressure gradient. Figure 3 demonstrated a magnified pressure gradient in the stenotic region due to the higher amplitude ratio. This increased pressure gradient, acting as the driving force for flow, necessitates a corresponding rise in velocity to maintain the same flow rate through the narrower stenosis, hence the observed velocity augmentation. Conversely, the elevated amplitude ratio in aneurysms from Fig. 3 translates to a diminished pressure gradient. This, coupled with the reduced resistance offered by the wider channel, translates to a lower driving force for the flow in the aneurysm. Consequently, the velocity decreases with increasing aneurysm size as the same flow rate needs to be distributed across a larger cross-sectional area, leading to a reduction in local fluid velocity. Figure 9 illustrates the relationship between the Hartmann number and velocity. With an increase in the Hartmann number, the centreline velocity decreases, leading to a rise in near-wall velocity due to mass flow rate conservation. Consequently, applying an external magnetic field leads to a flattened velocity profile near the centreline, resulting in a reduced rate of velocity change. This phenomenon is attributed to the induction of the Lorentz force, which decelerates the fluid motion. These observed patterns, as discussed^18^, indicate a potential reduction in blood velocity during surgical procedures. Figure 10 depicts the influence of the Hartmann number on velocity for fluid flow with two scenarios, pure blood ($\varphi =0$) and nano-blood ($\varphi =0.15$); the observed behaviour can be attributed to distinct physical mechanisms. In the case of nano-blood, the lower decreasing rate of velocity compared to pure blood suggests that nanoparticles contribute to a more stabilized flow. Practically, the observed lower rate of velocity decrease in the nanofluid scenario implies a potential advantage. It suggests that utilizing nanofluids in medical procedures might result in a more controlled and stable blood flow environment during surgery. This is a valuable consideration for optimizing procedures and ensuring patient safety^71^. In contrast, Fig. 11 presents a contrasting trend concerning the impact of porosity on velocity. It demonstrates that with an increase in the Darcy number, there is a corresponding growth in centreline velocity, coupled with a decline in near-wall velocity.

**Figure 12:**
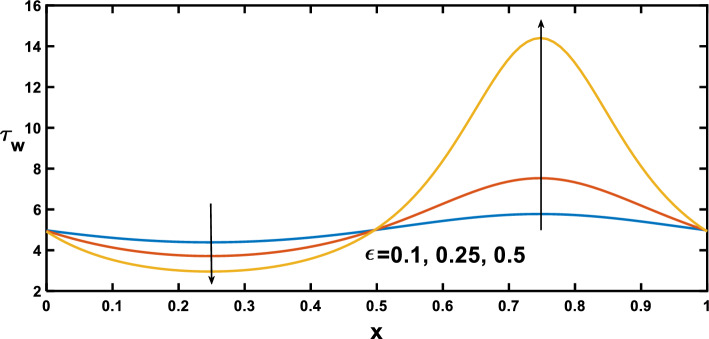
Wall shear stress profile for various values of the amplitude ratio $\left(\varepsilon \right)$.

**Figure 13:**
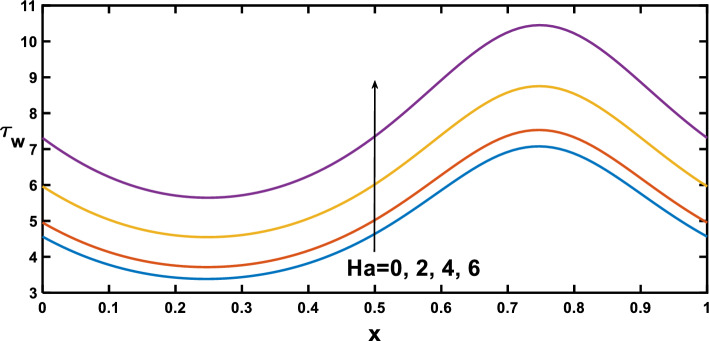
Wall shear stress profile for various values of Hartmann number $\left(Ha\right)$.

**Figure 14:**
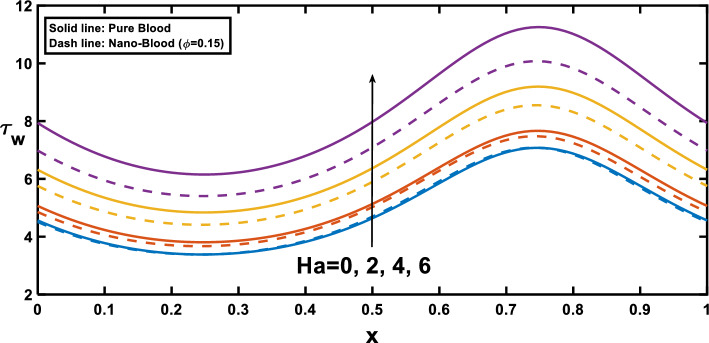
Wall shear stress profile for various values of Hartmann number $\left(Ha\right)$ and the nanoparticle concentration $(\varphi )$.

**Figure 15:**
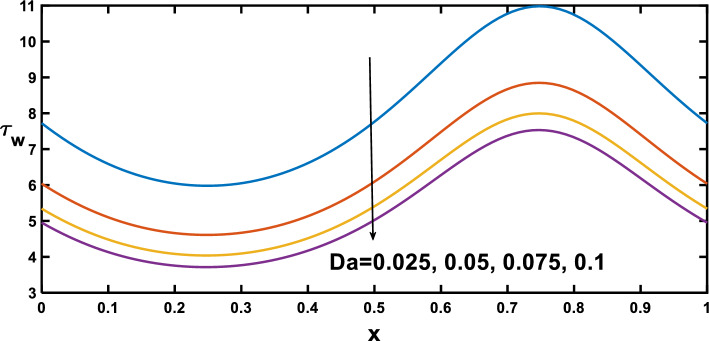
Wall shear stress profile for various values of the Darcy number $\left(Da\right)$.

The distribution of wall shear stress along the longitudinal direction of the channel reveals significant findings at the stenosed and aneurysm portions of the channel, demonstrated in Figs. 12, 13, 14 and 15, with different rheological parameters. Building upon Fig. 3, 8), Fig. 12 reveals a critical interplay between shear stress, pressure gradients, and channel geometry in stenotic and aneurysmal conditions. It demonstrates elevated shear stress near the stenotic region and reduced shear stress near the aneurysm. This behaviour can be explained by considering velocity gradients. The narrowed channel in stenosis leads to higher velocity gradients, resulting in elevated shear stress near the stenosis. This aligns with the steeper pressure gradient observed in Fig. 3, as a steeper gradient necessitates a more significant driving force that needs to be overcome by the increased shear stress for flow maintenance. Conversely, the wider channel in an aneurysm leads to reduced velocity gradients, translating to lower shear stress near the aneurysmal region. This aligns with the lower pressure gradient in Fig. 3, as a lower gradient signifies a smaller driving force that can be balanced by the lower shear stress in the aneurysm. These observations are consistent with findings in Ref. ^53^ highlighting the crucial role of geometry in influencing shear stress patterns. Figure 13 provides significant insights, demonstrating that as the Hartmann number increases, the slope of the velocity profile near the wall also rises. This increased slope, as depicted in Fig. 9, leads to a corresponding elevation in wall shear stress, which closely aligns with^16^. Figure 14 illustrates the impact of the Hartmann number on wall shear stress for fluid flow in two scenarios pure blood: $(\varphi =0)$ and nano-blood $(\varphi =0.15)$. Notably, lower wall shear stress was observed in the case of nano-blood compared to pure blood. Figure 15 illustrates a decrease in the shear stress at the wall with an increasing Darcy number. This reduction can be attributed to the diminishing slope of the velocity profile near the wall, as vividly demonstrated in Fig. 11.

**Figure 16:**
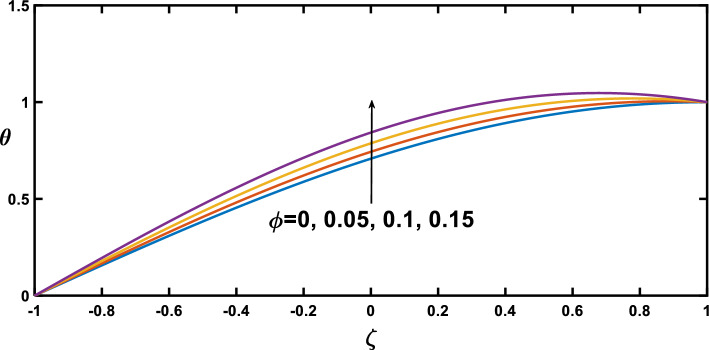
Temperature profile for various values of the nanoparticle concentration $(\varphi )$.

**Figure 17:**
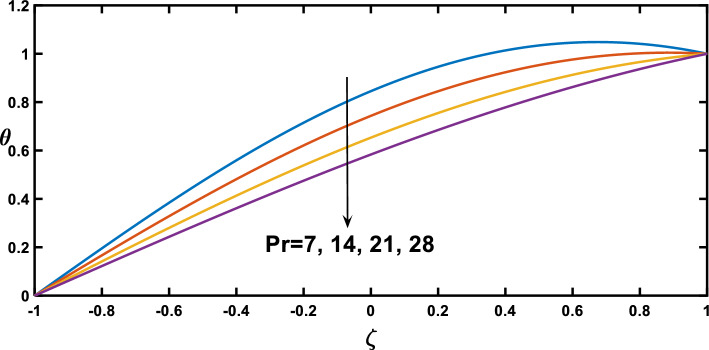
Temperature profile for various values of Prandtl number $(Pr)$.

**Figure 18:**
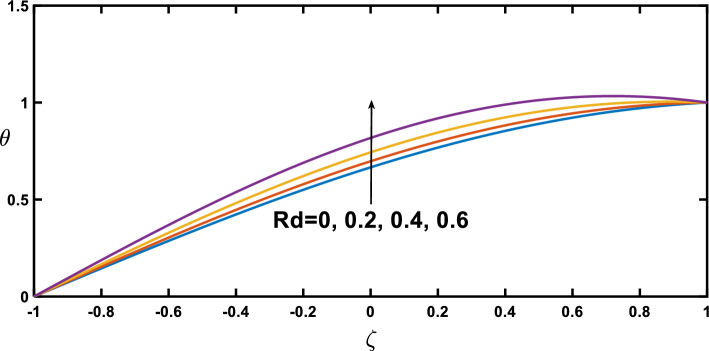
Temperature profile for various values of the radiation parameter $(Rd)$.

**Figure 19:**
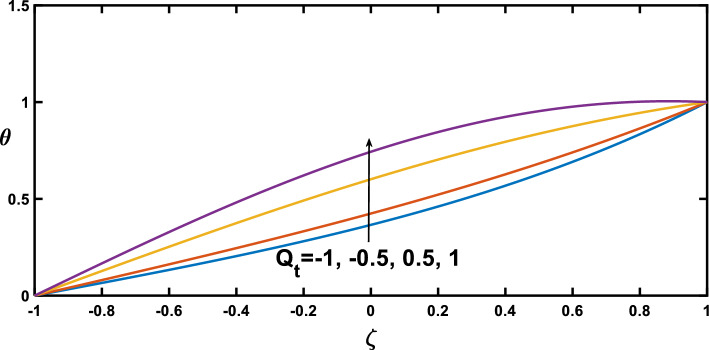
Temperature profile for various values of the heat source/sink $({Q}_{t})$.

Regarding the temperature profiles, as depicted in Fig. 16, as the volume fraction of nanoparticles increases, the surface area is substantially enlarged to blood flow, facilitating enhanced heat transfer within the fluid, and this result closely agrees with^10^. A higher Prandtl number signifies a lower thermal diffusivity relative to momentum diffusivity, resulting in less efficient heat conduction than momentum transfer. This leads to a lower temperature profile with an increasing Prandtl number, as illustrated in Fig. 17. Figure 18 demonstrates that temperature increases as the radiation parameter grows, highlighting the significance of radiative heating. Therefore, with a higher radiation parameter, the fluid receives more thermal energy through radiation, leading to an overall increase in temperature. This aligns with the discussion in Ref.^32^, emphasizing the significance of radiative heating in nanofluid flow scenarios. The temperature profile for different values of ${Q}_{t}$ (heat source) is plotted in Fig. 19, revealing an enhancement in temperature significance with increasing ${Q}_{t}$. As the heat source rises, the heat input from nanoparticles intensifies, contributing to a notable rise in temperature, and this result closely aligns with^53^. This enhanced temperature is primarily due to the effective thermal properties of nanoparticles, which facilitate heat conduction or absorption. In the context of blood flow applications involving nanoparticles, this phenomenon carries practical significance. For example, in hyperthermia treatments, controlled heating targets specific areas for therapeutic purposes^72^.

**Figure 20:**
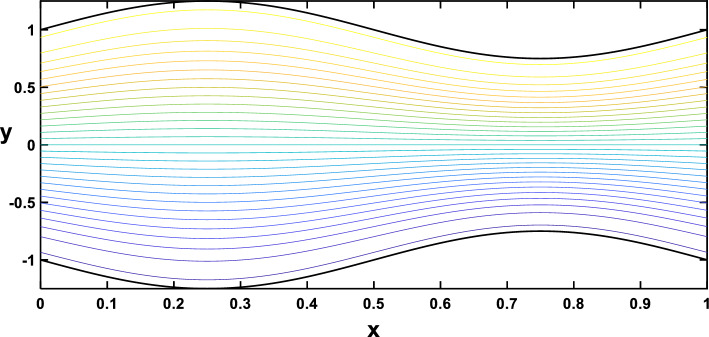
Streamlines for different values of the time $\left(t=\frac{\pi }{4}\right)$.

**Figure 21:**
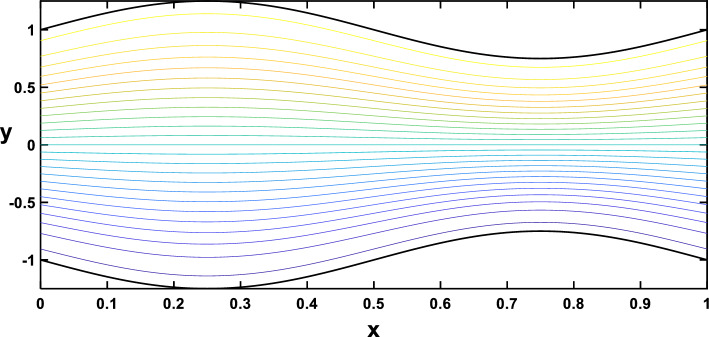
Streamlines for different values of the time $\left(t=\frac{\pi }{2}\right)$.

**Figure 22:**
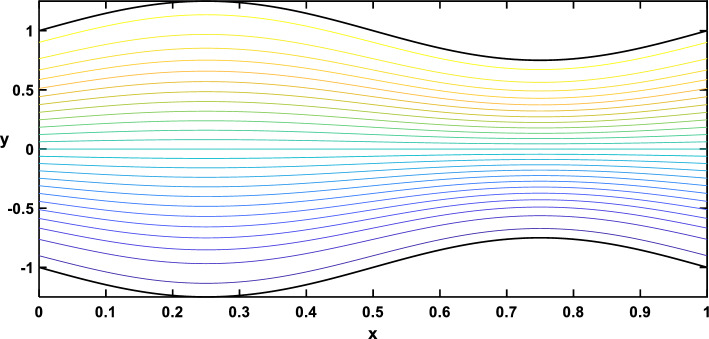
Streamlines for different values of the Hartmann number $\left(Ha=0\right)$.

**Figure 23:**
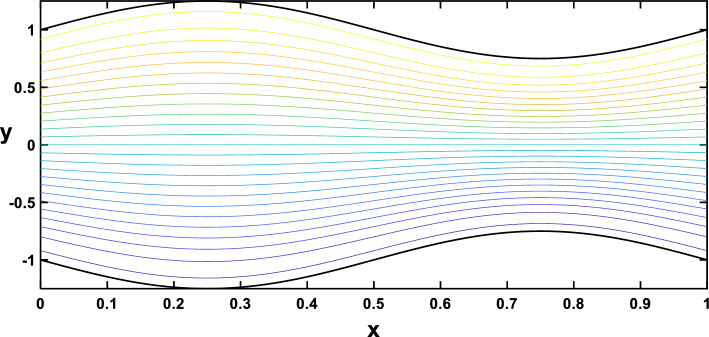
Streamlines for different values of the Hartmann number $\left(Ha=6\right)$.

In Figs. 20, 21, 22, 23, streamlines for different values of the time and the Hartmann number are examined. With the increase in the time and the Hartmann number, it is observed that the streamlines become more separated. These observations hold significant practical implications. It is indicated that the flow patterns within the channel are influenced by the strength of the magnetic field, represented by the Hartmann number. The increased separation between streamlines implies a more controlled and directed flow, which may find applications in microfluidics and drug delivery.

**Figure 24:**
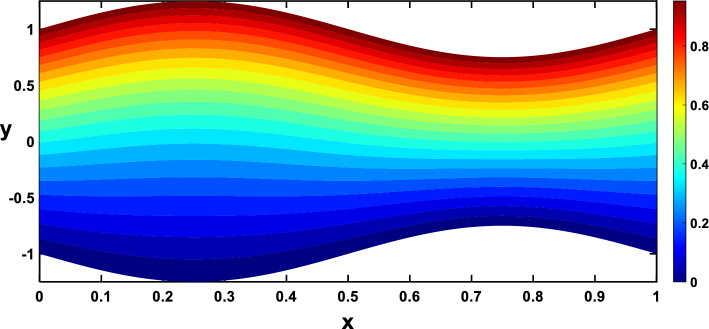
Isothermal lines for different values of the nanoparticle concentration $(\varphi =0)$.

**Figure 25:**
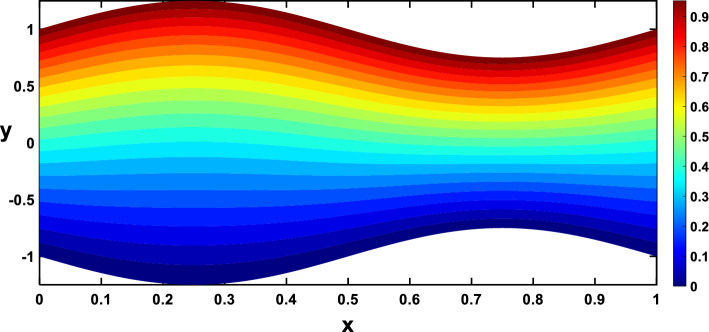
Isothermal lines for different values of the nanoparticle concentration $(\varphi =0.15)$.

**Figure 26:**
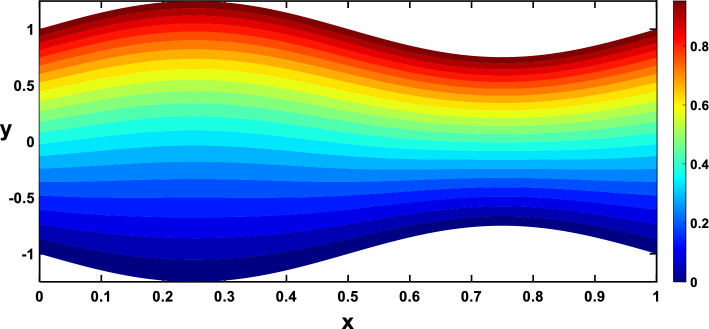
Isothermal lines for different values of the radiation parameter $(Rd=0)$.

**Figure 27:**
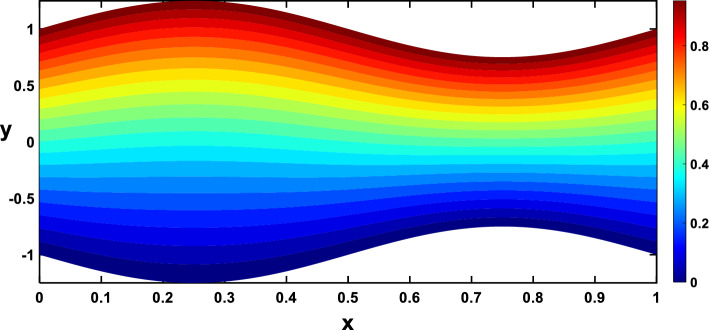
Isothermal lines for different values of the radiation parameter $(Rd=0.6)$.

**Figure 28:**
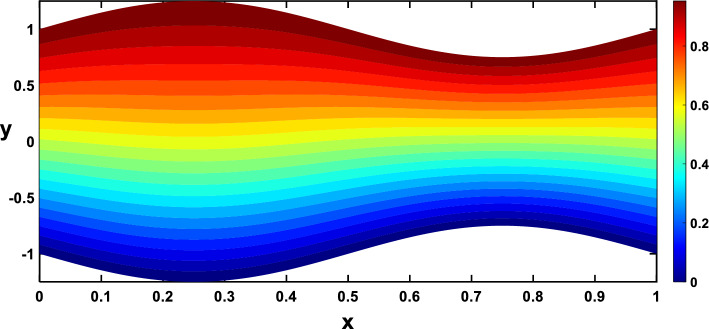
Isothermal lines for different values of the heat source $({Q}_{t}=-0.5)$.

**Figure 29:**
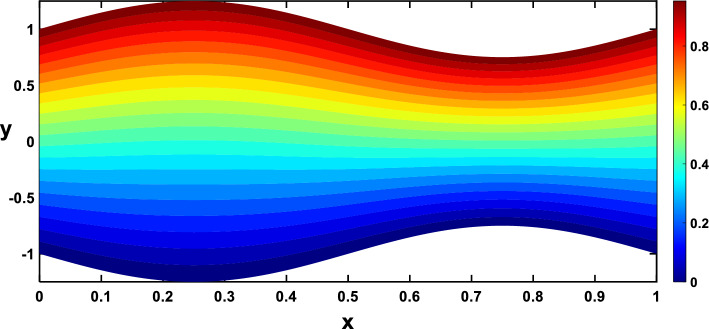
Isothermal lines for different values of the heat source $({Q}_{t}=0.5)$.

Nanoparticles often have higher thermal conductivities compared to the base fluid. As the concentration of nanoparticles increases, the overall thermal conductivity of the nanofluid also increases. This means heat can transfer more readily through the fluid, leading to a higher temperature gradient and, consequently, more divergent isothermal lines, as shown in Figs. 24, 25. In Figs. 26, 27, The radiation parameter represents the relative importance of radiative heat transfer compared to conductive heat transfer within the fluid. As the radiation parameter increases, radiative heat transfer becomes more dominant. This allows heat to travel through the fluid via electromagnetic waves, potentially creating deeper penetration and more localized heating than pure conduction. Consequently, temperature gradients increased, leading to more divergent isothermal lines. A stronger heat source directly pumps more thermal energy into the fluid, leading to higher local temperatures around the source. This creates steeper temperature gradients in the surrounding regions, causing the isothermal lines to spread further apart to reflect these variations, as illustrated in Figs. 28, 29.

## Conclusion

In summary, this current work delves into the behaviour of unsteady pulsatile nano-blood flow in a two-dimensional porous wavy channel with both aneurysm and stenosis, considering the impact of a magnetic field and heat transfer. The mathematical model incorporates nonlinear partial differential equations solved using the perturbation technique, and the influence of pertinent parameters is discussed. The results have been validated and are in good agreement with those in the literature. The main findings from the graphical representations can be summarized as follows:

- In stenotic regions, as the size of the stenosis grows, the pressure gradient rises, whereas the aneurysmal segment has the opposite trend. Moreover, the pressure gradient variation is lower in aneurysm segments than in stenosis segments.
- A significant increase in velocity is observed as the size of the stenosis grows, with a decrease as the size of the aneurysm increases. Additionally, the velocity profile decreases with a rise in the magnetic field at the centreline, while it increases with an increase in the permeability parameter.
- In the presence of a magnetic field, pure blood has a higher velocity than nano-blood flow.
- The shear stress at the wall in the stenosis segment grows to its maximum before dropping dramatically and bottoming out near the end of the stenosis section. In contrast, the aneurysmal segment has the opposite trend. The wall shear stress profile increases with an increase in the magnetic field. It is also apparent that the wall shear stress decreases as the permeability parameter increases.
- The temperature profile increases with increasing magnetic field, nanoparticle volume fraction, heat source, and radiation parameter but decreases with the permeability parameter and Prandtl number rising.

This study’s findings extend beyond theoretical insights, holding significant potential for real-world applications across diverse industries. In biomedical engineering, understanding pressure variations in diseased blood vessels can inform the design of safer and more effective medical devices for vascular interventions. Similarly, analysing velocity profiles under magnetic fields can guide the development of improved MRI technologies and magnetic drug targeting systems. Characterizing shear stress profiles at vessel walls offers invaluable insights for the cardiovascular device industry, enabling them to design implants, stents, and other devices that minimize risks to blood flow and vascular health.

### Supplementary Information

Supplementary Information.

## Data Availability

The datasets used and/or analysed during the current study available from the corresponding author on reasonable request.

## Abbreviations

${x}^{*}$: Dimensional coordinate along the channel $\left(m\right)$
${y}^{*}$: Dimensional coordinate perpendicular to the channel $\left(m\right)$
$$x, y$$: Dimensionless distances
$${t}^{*}$$: Dimensional time $$\left(s\right)$$
$$t$$: Dimensionless time
$${p}^{*}$$: Pressure $$\left(kg/m{ .s}^{2}\right)$$
${u}^{*}$: Dimensional velocity in X-direction $\left(m/s\right)$
${v}^{*}$: Dimensional velocity in Y-direction $\left(m/s\right)$
$u$: Dimensionless velocity in X-direction
$v$: Dimensionless velocity in Y-direction
${T}^{*}$: Temperature of the nanofluid $\left(K\right)$
${T}_{w}$: Temperature at the upper wall $\left(K\right)$
${T}_{0}$: Temperature at the lower wall $\left(K\right)$
$${q}_{r}$$: Heat flux $$\left(W/{m}^{2}\right)$$
${k}_{f}$: Thermal conductivity of the fluid $\left(W/m .K\right)$
${k}_{n}$: Thermal conductivity of the nanoparticles $\left(W/m .K\right)$
${k}_{nf}$: Thermal conductivity of the nanofluid $\left(W/m .K\right)$
$k$: Permeability of the porous media $\left({m}^{2}\right)$
$a$: Height of the wall constriction $\left(m\right)$
$d$: Half width of the channel $\left(m\right)$
${B}_{0}$: Uniform magnetic field $\left(T\right)$
${k}^{*}$: Rosseland mean absorption coefficient $\left({m}^{-1}\right)$
$Da$: Darcy number of the porous media
$$Ha$$: Hartmann number
$$Pr$$: Prandtl number
$$Rd$$: Radiation parameter
$Q$: Volumetric flow rate $\left({m}^{3}/s\right)$
${Q}_{0}$: Heat source/sink parameter $\left(W/{m}^{2}\right)$

## Greek symbols

${\mu }_{nf}$: Dynamic viscosity of the nanofluid $\left(kg/m.s\right)$
${\rho }_{nf}$: Density of the nanofluid $\left(kg/m.s\right)$
${\upsilon }_{nf}$: Kinematic viscosity of the nanofluid $\left({m}^{2}/s\right)$
${\mu }_{f}$: Dynamic viscosity of the fluid $\left(kg/m.s\right)$
${\rho }_{f}$: The density of the fluid $\left(kg/m.s\right)$
${\upsilon }_{f}$: Kinematic viscosity of the fluid $\left({m}^{2}/s\right)$
${\rho }_{n}$: Density of the nanoparticles $\left(kg/m.s\right)$
${\left(\rho {C}_{p}\right)}_{f}$: Heat capacity of the fluid $\left(kg/m.{s}^{2}.K\right)$
${\left(\rho {C}_{p}\right)}_{n}$: Heat capacity of the nanoparticles $\left(kg/m.{s}^{2}.K\right)$
${\left(\rho {C}_{p}\right)}_{nf}$: Heat capacity of the nanofluid $\left(kg/m.{s}^{2}.K\right)$
$\eta$: Height of an abnormal channel $\left(m\right)$
$\lambda$: Length of wall constriction $\left(m\right)$
$\varepsilon$: Amplitude ratio parameter $\left(m\right)$
$\delta$: Wall slope parameter
$\sigma$: Electrical conductivity of the fluid $\left(S/m\right)$
${\sigma }^{*}$: Stefan-Boltzmann constant $\left(W/{m}^{2}. {K}^{4}\right)$
$\varphi$: Nanoparticle volume fraction
$\omega$: The angular frequency of the flow $\left({s}^{-1}\right)$
$\psi$: Dimensionless streamlines function
$$\theta$$: Dimensionless temperature
${\tau }_{w}$: Wall shear stress
$\zeta$: Dimensionless transverse coordinate

## Subscripts

$$f$$: Fluid fraction
$$n$$: Nanoparticle
$$nf$$: Nanofluid fraction
