# Supplementary material for: Pulsatile nanofluid flow with variable pressure gradient and heat transfer in wavy channel
Source: Sci Rep. 2024 Apr 23;14:9351. doi: 10.1038/s41598-024-59251-9 (PMC11039767; doi:10.1038/s41598-024-59251-9)
Supplement: Supplementary file 1 — Supplementary Information. [file 41598_2024_59251_MOESM1_ESM.docx]

**Appendix**

| $B_{1}=(1-\varphi)^{2.5}\left( 1-\varphi+\left( \frac{\varphi\rho_{n}}{\rho_{f}} \right) \right),$  $B_{2}=\frac{Pr \left( 1-\varphi+\left( \frac{\varphi\left( \rho C_{p} \right)_{n}}{\left( \rho C_{p} \right)_{f}} \right) \right)}{\left( \left( \frac{k_{nf}}{k_{f}} \right)+\left( \frac{4}{3} \right)Rd \right)},$  $m=\sqrt{\left( Ha^{2}(1-\varphi)^{2.5}+\frac{1}{Da} \right)},$  $m_{1}=\sqrt{\frac{Q_{t} Pr}{\left( \left( \frac{k_{nf}}{k_{f}} \right)+\left( \frac{4}{3} \right)Rd \right)}},$  $C_{1}=\frac{1}{\sinh\left( m\eta\right)-\eta m\cosh\left( m\eta\right)},$  $C_{2}=i B_{1}m^{2}Q C_{1},$  $C_{3}=\frac{\eta}{m^{2}}-\frac{\sinh\left( 2m\eta\right)}{2m^{3}},$  $C_{4}=B_{1}Q^{2} m^{5} C_{1}^{3}\frac{d\eta}{dx}\sinh\left( m\eta\right),$  ${C_{5}=C}_{1}\left[ \sinh\left( m\eta\right)\left( \frac{2\eta\cosh\left( m\eta\right)}{m^{2}}-\frac{2\sinh\left( m\eta\right)}{m^{3}}-\frac{\eta^{2}\sinh\left( m\eta\right)}{m}+\frac{\eta^{3}\cosh\left( m\eta\right)}{4} \right)-\eta\cosh\left( m\eta\right)\left( \frac{\eta^{2}\sinh\left( m\eta\right)}{2}+\frac{\sinh\left( m\eta\right)}{m^{2}}-\frac{\eta\cosh\left( m\eta\right)}{m}+\frac{kn m \eta^{2} \cosh\left( m\eta\right)}{2} \right) \right],$  $C_{6}=\sinh\left( m\eta\right)\left( \frac{5\cosh\left( m\eta\right)}{4m^{4}}-\frac{2 sinh \left( m\eta\right)}{\eta m^{5}}-\frac{\eta\sinh\left( m\eta\right)}{4m^{3}} \right)-\eta\cosh\left( m\eta\right)\left( \frac{\sinh\left( m\eta\right)}{\eta m^{4}}-\frac{\cosh\left( m\eta\right)}{2m^{3}} \right),$  $C_{7}=\frac{B_{2}Q m_{1}m^{2} C_{1}^{2}\frac{d\eta}{dx}\sinh\left( m\eta\right)}{2},$  $C_{8}=\frac{-1}{\cos\left( m_{1}\eta\right)} \left[ C_{12}\frac{\sin\left( m_{1}\eta\right)\sinh\left( m\eta\right)}{m(m^{2}+4m_{1}^{2})}+C_{13} \frac{\cos\left( m_{1}\eta\right)\cosh\left( m\eta\right)}{m(m^{2}+4m_{1}^{2})}+C_{16}\frac{\eta\sin\left( m_{1}\eta\right)}{4m_{1}^{2}}+C_{19}\frac{\eta^{2}\cos\left( m_{1}\eta\right)}{4m_{1}} \right],$  $C_{9}=\frac{-1}{\sin\left( m_{1}\eta\right)} \left[ C_{14} \frac{\cos\left( m_{1}\eta\right)\sinh\left( m\eta\right)}{m(m^{2}+4m_{1}^{2})}+C_{15} \frac{\sin\left( m_{1}\eta\right)\cosh\left( m\eta\right)}{m(m^{2}+4m_{1}^{2})}+C_{17}\frac{\eta\cos\left( m_{1}\eta\right)}{4m_{1}^{2}}+C_{18}\frac{\eta^{2}\sin\left( m_{1}\eta\right)}{4m_{1}} \right],$  $C_{10}=\frac{2m_{1}\sin(m_{1}\eta)}{C_{1}m\cos^{2} \left( m_{1}\eta\right) \sinh(m\eta)}+\frac{m \eta}{cos(m_{1}\eta)},$  $C_{11}=\frac{sin(m_{1}\eta)}{C_{1}\cos^{2} \left( m_{1}\eta\right) \sinh(m\eta)}-\frac{2m_{1}\eta}{\cos\left( m_{1}\eta\right)},$  $C_{12}=\frac{2m_{1}\cos(m_{1}\eta)}{C_{1}m\sin^{2} \left( m_{1}\eta\right) sinh(m\eta)}-\frac{m \eta}{\sin\left( m_{1}\eta\right)},$  $C_{13}=-\frac{\cos\left( m_{1}\eta\right)}{C_{1}\sin^{2} \left( m_{1}\eta\right)\sinh\left( m\eta\right)}-\frac{2m_{1}\eta}{\sin\left( m_{1}\eta\right)},$  $C_{14}=-\frac{2m_{1}\sin\left( m_{1}\eta\right)\cosh\left( m\eta\right)}{C_{1}m\cos^{2} \left( m_{1}\eta\right)\sinh\left( m\eta\right)}-\frac{\sinh\left( m\eta\right)}{\cos\left( m_{1}\eta\right)},$  $C_{15}=-\frac{2m_{1}\cos\left( m_{1}\eta\right)\cosh\left( m\eta\right)}{C_{1}m\sin^{2} \left( m_{1}\eta\right)\sinh\left( m\eta\right)}+\frac{\sinh\left( m\eta\right)}{\sin\left( m_{1}\eta\right)},$  $C_{16}=\frac{\sinh\left( m\eta\right)}{\sin(m_{1}\eta)},$  $C_{17}=\frac{\sinh\left( m\eta\right)}{\cos(m_{1}\eta)}.$ |
| --- |
